# Momentary Induction of Inhibitory Control and Its Effects on Uncertainty

**DOI:** 10.5334/joc.133

**Published:** 2021-01-21

**Authors:** Omer Linkovski, Carolyn I. Rodriguez, Michael G. Wheaton, Avishai Henik, Gideon E. Anholt

**Affiliations:** 1 Department of Psychology, Bar-Ilan University, Ramat-Gan, Israel; 2 Department of Medical Neurobiology, Hebrew University School of Medicine, Jerusalem, Israel; 3 Department of Psychiatry and Behavioral Sciences, Stanford University Medical C, Stanford, CA, USA; 4 Veterans Affairs Palo Alto Health Care System, Palo Alto, CA, USA; 5 Department of Psychology, Barnard College, Columbia University, New York, NY, USA; 6 Department of Psychology, Ben-Gurion University of the Negev, Beer Sheva, Israel; 7 Zlotowski Center for Neuroscience, Ben-Gurion University of the Negev, Beer Sheva, Israel

**Keywords:** compulsions, uncertainty, inhibitory control, Stroop task, action tendencies, Obsessive-Compulsive Disorder

## Abstract

Deficient inhibitory control and difficulty resolving uncertainty are central in psychopathology. How these factors interact remains unclear. Initial evidence suggests that inducing inhibitory control improves resolution of uncertainty. This may occur only when participants overcome action tendencies, which are dominant tendencies to perform certain behaviors. Our study explored the links between inhibitory control and behavioral responses to uncertainty while manipulating action-tendencies’ strength. In three experiments, 132 undergraduates completed a task that combined induction of momentary changes in inhibitory control level (Stroop task), with responses to uncertainty (visual-search task). We manipulated action-tendencies’ strength by varying uncertainty proportions across experiments. Results indicated that momentary induction of inhibitory control improved resolution of high-uncertainty during mostly low-uncertainty trials but hampered resolution of low-uncertainty during mostly high-uncertainty trials. Identical inhibitory control induction did not affect resolution of uncertainty when uncertainty conditions were equalized. Participants’ subjective uncertainty measures were similar across experiments. Our results suggest that momentary inhibitory control induction modifies behavioral responses to uncertainty and selectively affects trials that require overcoming dominant action tendencies. These findings indicate a potentially unique and multifaceted relationship between inhibitory control and behavioral responses to uncertainty. Clinical implications for models of Obsessive-Compulsive Disorder and experimental implications to post-conflict processes are discussed.

## INTRODUCTION

In order to function adaptively, humans prioritize goal directed behaviors while controlling more habitual or immediate stimuli-driven actions and unwanted thoughts. Inhibitory control is defined as a set of mechanisms supporting goal-directed behavior while inhibiting and regulating intense predispositions, unwanted thoughts and behaviors (Diamond, 2013). Deficient inhibitory control is a trans-diagnostic factor in psychopathologies involving unwanted thoughts and behaviors (Goodkind et al., 2015; McTeague et al., 2016).

In service of this important function, dedicated neural circuits are activated when we regulate our attention and motor responses (Niendam et al., 2012). Interestingly, most of these circuits are also activated when regulating emotions (Ochsner et al., 2012). Several studies have reported that activating the inhibitory control system improves participants’ subsequent capacity to regulate behavioral and physiological responses to emotional stimuli or risky choices (Cohen, Moyal, et al., 2015; Verbruggen et al., 2013). Moreover, training inhibitory control reduces highly anxious students’ unwanted intrusive thoughts (Cohen, Mor, et al., 2015) and when combined with therapy, such training may even reduce clinical OCD symptoms (Kalanthroff et al., 2018). Importantly, inhibitory control training appears to strengthen functional connections between limbic brain regions, and central regions in the inhibitory control network (i.e., right inferior frontal gyrus)(Cohen et al., 2016). These studies suggest that inhibitory control may reduce emotional activation to negatively valenced images. Less is known about inhibitory control’s ability to regulate participants’ responses to different types of aversive phenomena such as uncertainty, which often breeds unwanted thoughts and behaviors (Shihata et al., 2016).

Uncertainty is induced by “the perceived absence of information at any level of consciousness or processing” (Carleton, 2016). Difficulty resolving or enduring uncertainty is a trans-diagnostic risk factor for psychopathologies involving unwanted thoughts and actions across obsessive-compulsive spectrum disorders (e.g., Obsessive Compulsive Disorder)(OCD), anxiety disorders (e.g., Generalized Anxiety Disorder) and trauma-related disorders (e.g., Post Traumatic Stress Disorder) (Boswell et al., 2013; Carleton et al., 2012; Gillett et al., 2018; Hong & Cheung, 2015; Kiverstein et al., 2019; McEvoy & Mahoney, 2012; Orban et al., 2003; Raines et al., 2019). Even mild uncertainty, caused by neutral stimuli, may elicit abnormal behavioral responses. For example, such uncertainty causes excessive checking in OCD patients compared with control participants (Toffolo et al., 2013, 2014). Using eye tracking in a visual search task (Treisman, 1982), where participants manually indicate whether a target stimulus is present or absent in an array of stimuli, Toffolo et al., found that OCD patients engage in more eye fixations than healthy controls only in trials featuring uncertainty – when the target line was absent (Toffolo et al., 2013, 2014) (for more details see current section 2.2.2.1).

While inhibitory control is considered a hallmark of top-down processes, its efficient deployment may depend on automatic bottom-up processes (Verbruggen et al., 2014). One important bottom-up process is action tendency, defined as the automatic predisposition to engage in the specific action an object evokes (e.g., reaching out and turning a door handle). Modifying action tendencies to clinically-relevant items has been shown to reduce avoidance and improve treatment outcomes in anxiety disorders and substance use disorders, both featuring unwanted thoughts and behaviors (Amir et al., 2008; Wiers et al., 2011). Moreover, OCD patients display enhanced action tendencies even to neutral stimuli (Dayan et al., 2014, 2017). However, to the best of our knowledge, no study related action tendencies to inhibitory control or to uncertainty.

Surprisingly little is known about the interaction between inhibitory control deficits and problems resolving uncertainty, particularly when considering that both have been suggested as trans-diagnostic processes (Linkovski et al., 2016; McTeague et al., 2016; Shihata et al., 2016). Uncertainty generally slows reaction times (RTs) in tasks (Hodsoll & Humphreys, 2001), including in tasks assessing inhibitory control (Sabri et al., 2001). More clinically relevant, efficient inhibitory control has been suggested to prevent development of uncertainty following repeated checking (Anholt et al., 2012; Linkovski et al., 2013). A direct inhibitory control manipulation speeded resolution of uncertainty in high-uncertainty trials (Kalanthroff et al., 2016). Building on Toffolo et al.’s findings, Kalanthroff et al., (2016) demonstrated that healthy students resolve uncertain situations faster in experimental blocks which included more frequent inhibitory control activation. However, Kalanthroff et al.’s study featured a single experiment, which included strong action tendencies, and compared inhibitory control induction between two experimental blocks. Kalathnhroff et al., (2016) did not modulate action-tendencies’ intensity and did not test whether activating inhibitory control can improve behavioral manifestation of uncertainty within a single trial. Modulating action tendencies while testing inhibitory control’s impact on behavioral manifestations of uncertainty may integrate top-down and bottom-up accounts of unwanted thoughts or behaviors. Since most psychological models of unwanted-repetitive thoughts and behaviors describe a vicious cycle in which an unwanted cognition increases one’s distress, strengthening urges for an unwanted action, an ecologically valid experimental procedure may inform our mechanistic understanding of unwanted thoughts and behaviors.

The current study explores the interaction between behavioral manifestation of uncertainty and inhibitory control by testing how inducing inhibitory control affects resolution of high and low uncertainty under different levels of action tendency. We manipulated differential action tendencies by modifying the proportions of trial types and their corresponding actions across three experiments as described below. We hypothesized that momentary inhibitory control induction with a different task from Kalanthroff et al., (2016), would lead to faster RTs in high uncertainty situations. With respect to action tendencies, we anticipated that the effect of inhibitory control on the behavioral manifestation of uncertainty would be larger when overcoming action tendencies.

## 1. EXPERIMENT 1

### 1.1. INTRODUCTION

Kalanthroff et al., (2016) demonstrated that inducing inhibitory control affects resolution of high-uncertainty trials over an experimental block. Inducing inhibitory control over an entire experimental block precluded conclusions concerning the effect of momentary fluctuations in levels of inhibitory control on uncertainty. The current experiment uses a different experimental design and task to induce momentary inhibitory control, extending validity of previous work (Kalanthroff et al., 2016).

### 1.2. MATERIALS AND METHODS

#### 1.2.1. Participants

Forty-five healthy participants, recruited via Ben-Gurion University’s online experiments system, participated for course credit or a small monetary amount. The university’s institutional review board approved the experiment and written informed consent was obtained from each participant. All participants were righthanded, had normal or corrected-to-normal vision, had no self-reported history of attention deficit disorder or dyslexia, and all were naive as to the purpose of the experiment. Four participants were excluded from further analysis – One participant due to misunderstanding the task, one reported that he made several purposeful errors as he\she predicted the study has a different purpose than the explained one, and two participants were excluded due to having extreme number of errors (i.e., above 2.5 standard deviations (SD) from the mean). Thus, 41 participants were included in the following analyses (28 females and 13 males, (mean age; M = 23.46 years, SD = 1.12).

#### 1.2.2. Apparatus

Data collection and stimuli presentation were controlled by a DELL OptiPlex 760 vPro computer with an Intel core 2 duo processor E8400 3 GHz. Stimuli were presented on a DELL E198PF 19″ LCD monitor. Participants were tested individually and sat approximately 24 in. from the computer screen. A keyboard was placed on a table between the participant and the computer monitor. E-prime software was used for programming, presentation of the stimuli and timing operations.

#### 1.2.3. Tasks

##### 1.2.3.1. Stroop task

We induced momentary inhibitory control using the Stroop task, in which participants are required to indicate the ink color a word is printed in (i.e., red, blue) while disregarding information about the meaning of the word (Kleiman et al., 2016; MacLeod, 1991). Incongruent color words (e.g., ‘red’ written in blue ink) require inhibiting the semantic information, which is achieved by a stronger activation of the brain’s inhibitory control system compared to congruent color words (i.e., ‘red’ written in red ink) (Roberts & Hall, 2008).

##### 1.2.3.2. Visual search task

The visual search task (Treisman, 1982) was used to experimentally induce mild uncertainty (Toffolo et al., 2013). In this task, participants indicate whether a target stimulus appears within a given array. The target stimulus may be present or absent in a given trial. While responses to target-present trials rely on the existence of an external stimulus, responses to target-absent trials rely solely on an internal criterion, as no external stimulus is present. Indeed, target-absent trials induce more uncertainty than target-present trials (Toffolo et al., 2014). Target-absent trials also induce more checking behaviors (i.e., eye-fixations) than target-present trials in OCD patients and in participants with subclinical obsessive-compulsive symptoms (Toffolo et al., 2013, 2014). Our current experiments assess RTs in the visual search task since previous experiments found participants’ checking behaviors were highly correlated with RTs (Toffolo et al., 2013, 2014). In the current experiments, participants responded by pressing one key in target-present trials and another in target-absent trials with the stimuli randomly dispersed across the computer monitor. The target line in the visual-search task was a 1.4 cm long green line, at a 45° incline. Non-target lines differed in either color (pink, gray) or orientation (vertical, 135° incline), so that all lines shared one feature with the target (i.e., orientation or color).

#### 1.2.4. Procedure

**Figure 1:**
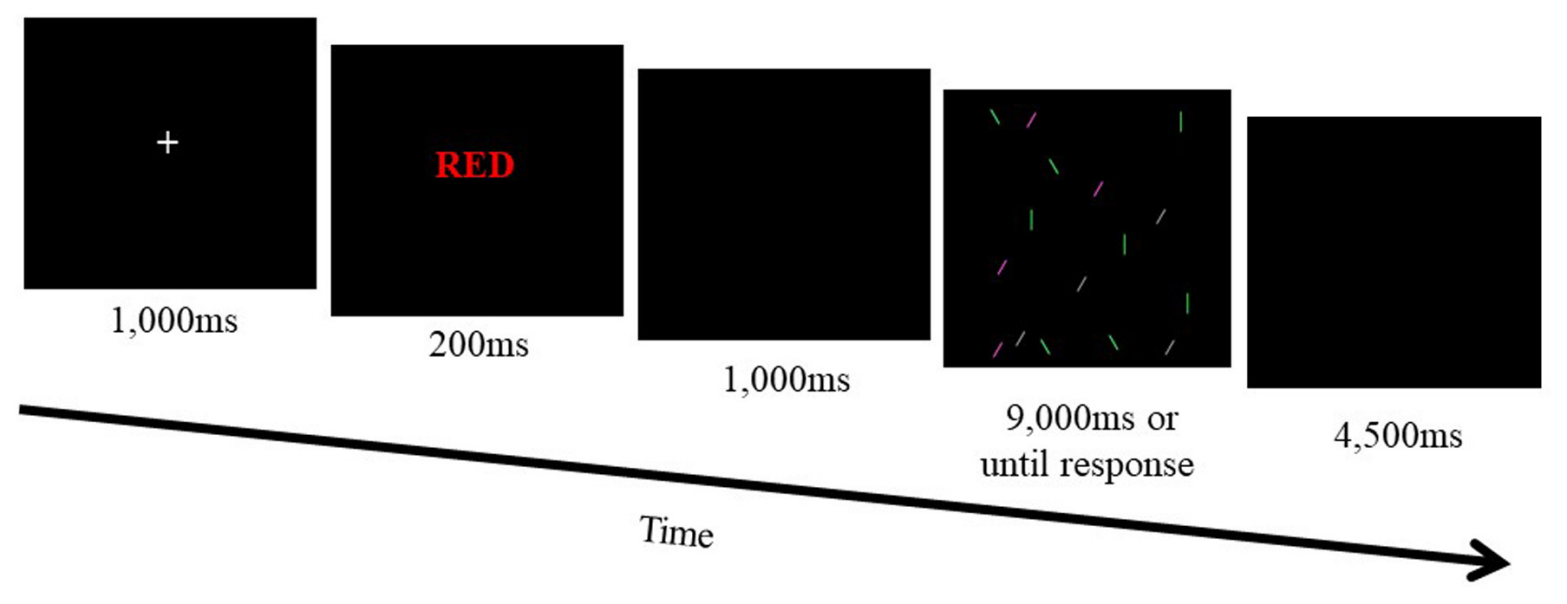
Example of a congruent Stroop trial followed by a target-absent trial. The target was a 1.4 cm long green line, slanted at 45°.

Participants performed a novel task interlacing the Stroop task with the visual search task (***Figure 1***). A trial began with a fixation cross appearing in the center of the screen for 1,000 milliseconds (ms). The fixation was followed by the presentation of a color word (i.e., Stroop task) for 200 ms. Participants manually indicated a word’s ink color (i.e., blue, red) within 3,000 ms by pressing either “m” or “,”. The word’s ink color was either congruent or incongruent to the meaning of the word (i.e., red, blue). For example, a congruent trial would feature the word ‘red’ printed in red ink, while an incongruent trial would feature the word ‘red’ printed in blue ink. Once participants responded, a blank screen appeared for 1,000 ms. Then, a visual search array appeared for 9,000 ms or until a response was made. All arrays featured 16 lines, differing in color (i.e., pink, grey, green) and in orientation (i.e., 0, 45, 135 degrees). In each trial participants pressed one of two keys (“z”, “x”) to indicate whether a green line, slanted in 45 degrees, was present or absent in the given array. All trials ended with an inter-trial interval (ITI) of 4,500 ms. The task featured 15 practice trials and 2 experimental blocks of 120 experimental trials per block. Experimental trials included an equal number of congruent and incongruent trials, but we skewed the distribution of the visual-search trials so that 80% of trials featured target-present trials and only 20% featured target-absent trials. Accordingly, there were 96 congruent Stroop trials followed by a target-present condition, 96 incongruent Stroop trials followed by a target-present condition, 24 incongruent Stroop trials followed by a target-absent condition and 24 congruent Stroop trials followed by a target-absent condition. Upon completion of the task participants were asked to rate their certainty with respect to their choices in target-absent and in target-present trials on a scale of zero (i.e., I was completely unsure about my choices) to a hundred (i.e., I was completely sure of my choices).

#### 1.2.5. Statistical analysis

We calculated RTs for correct responses in both the visual search segments and the Stroop segments of the task. Accuracy was computed as the proportion of correct results from all trials. As a manipulation check, testing whether the Stroop task affected inhibitory control, we compared RTs of congruent vs. incongruent Stroop trials using a paired-samples t-test. We then analyzed the effect of inhibitory control on the behavioral manifestation of uncertainty using separate two-way analyses of variance (ANOVAs) on RT and accuracy of the visual search task, with momentary level of inhibitory control (congruent and incongruent trials) and level of uncertainty (low, high) serving as within-participant factors. In order to investigate the interaction between uncertainty levels and inhibitory control induction, according to our a-priori assumption, we conducted two planned comparisons between high vs. low inhibitory control conditions, in target-absent and target-present conditions. Subjective certainty was analyzed using a paired-samples t-test, with visual-search trial type (target-absent, target-present) as a within participant factor. Two-tailed tests were used to examine our a-priori hypotheses.

A power analysis using G*Power 3.1 (Faul et al., 2007) based on previous effect sizes (Kalanthroff et al., 2016) indicated that the current sample allowed for examination of the two-way interaction between inhibitory control and uncertainty at a power > 95% to test medium to large size effects with a Type 1 error α < 0.05).

### 1.3. RESULTS

**Figure 2:**
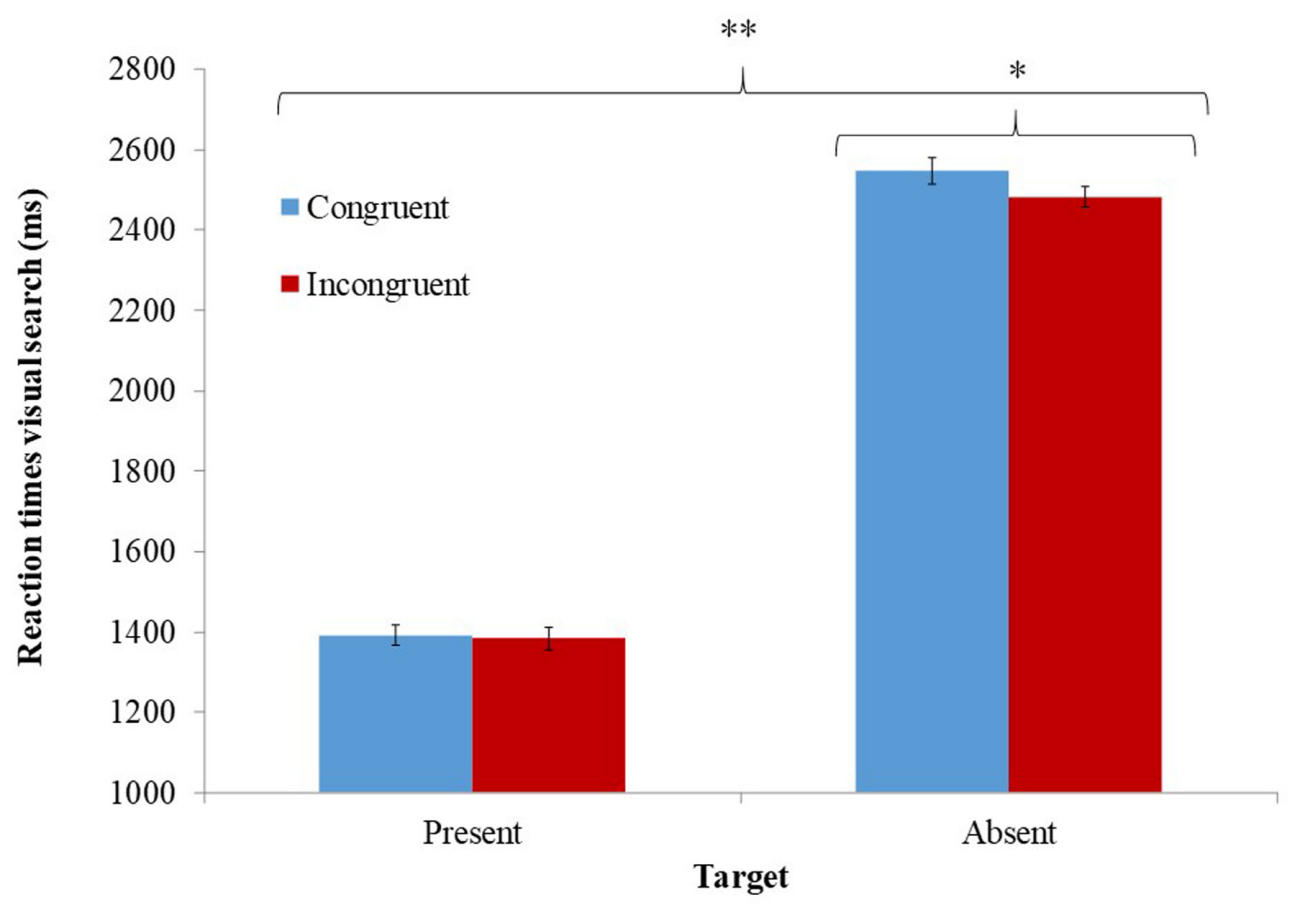
Mean RT in the different conditions of the visual search task as a function of the different inhibitory control conditions. * Significant at *p* < .05 levels. ** Significant at *p* < .0001 levels. Error bars represent ± 1 standard error (Cousineau, 2005).

We first analyzed RTs in the Stroop task as a manipulation check. A significant effect was evident for trial type *t*(40) = 5, *p* < .0001, *η^2^_p_* = .93 with slower RTs in incongruent trials (M = 652 ms, SD = 210) compared to congruent trials (M = 597 ms, SD = 163). Accuracy in the Stroop task did not significantly differ for congruent (M = .97, SD = .03) and incongruent trials (M = .96, SD = .04), *t*(40) = 1.83, *p* = .07, *η^2^_p_* = .078. The effect of Stroop trial type on RTs in the Stroop task indicates that congruent and incongruent trials induced different levels of inhibitory control. With respect to our a-priori hypothesis about interaction between inhibitory control and uncertainty, as expected, a two-way ANOVA revealed a significant main effect for uncertainty *F*(1, 40) = 495, *p* < .0001, *η^2^_p_* = .93, indicating slower RTs to target-absent trials (M = 2514 ms, SD = 566) compared to target-present trials (M = 1387 ms, SD = 311). There was also a significant two-way interaction between the inhibitory control induction and uncertainty *F*(1, 40) = 4.39, *p* < .05, *η^2^_p_* = .10 (***Figure 2***). Two planned comparisons further probed this interaction. Participants were significantly faster to resolve high-uncertainty trials following the momentary inhibitory control induction (i.e., after an incongruent Stroop trial; M = 2482 ms, SD = 547), compared to resolving high-uncertainty trials when their control system was not activated (i.e., after a congruent Stroop trial; M = 2547 ms, SD = 600), *F*(1, 40) = 5.03, *p* < .05. Inhibitory control induction did not modify resolution of low-uncertainty trials *F*(1, 40) = .17, *p* = .68. A similar analysis of participants’ accuracy in the visual search task did not detect effects of inhibitory control induction on uncertainty, *F*(1, 40) = 2.07, *p* = .16, *η^2^_p_* = .05, and *F*(1, 40) = .001, *p* = .98, for the two-way interaction and for the main effect of inhibitory control induction, respectively.

Subjective certainty of participants was significantly reduced (i.e., experienced higher uncertainty) in target-absent trials (M = 74.66, SD = 19) compared to subjective certainty in target-present trials (M = 94.54, SD = 5.7), F(1, 40) = 51.71, *p* < .0001, *η^2^_p_* = .56). As expected, target-absent trials were also subjectively rated as being high in uncertainty (i.e., high-rated uncertainty trials). *Figure S1* in the supplemental material available online presents a graphical summary of subjective certainty ratings.

### 1.4. DISCUSSION

Current results replicate and extend previous findings, suggesting that inducing inhibitory control can improve the behavioral manifestation of high-uncertainty situations. As expected, we obtained the selective effect of inhibitory-control induction on uncertainty, despite having used a different task from previous research. In contrast to our Stroop task, Kalanthroff et al., (Kalanthroff et al., 2016) used the stop-signal task. Successful resolution of either task requires inhibiting one’s response. Whereas the stop-signal requires participants to inhibit a response they have already started, the Stroop task requires participants to inhibit a response to irrelevant information (a word’s semantic meaning word) that is presented concurrently with the relevant information (the word’s color). Regardless of the task, inhibitory control affects behavioral manifestation of uncertainty.

It should be noted that in our present study and in Kalanthroff et al., (2016) there was an imbalance between target-present and target-absent trials, so that the dominant response was ‘present’. This imbalance between trial types increased the action tendency of the target-present response. An alternative explanation for the current results may be that inducing inhibitory control facilitates non-dominant responses. In order to ascertain action tendencies’ role in the interaction between inhibitory control and uncertainty, we replicate Experiment 1 while modifying proportions of target-present and target-absent conditions in experiment 2.

## 2. EXPERIMENT 2

### 2.1. INTRODUCTION

Experiment 2 will test the effect of inhibitory control induction on uncertainty when there is no dominant action tendency across visual search trials. Accordingly, current Experiment 2 will utilize the same task as Experiment 1 (***Figure 1***) but with equal proportions (i.e., 50%) of target-present and target-absent trials, so that the visual search task will have no dominant response. Current Experiment 2 will improve our ability to speculate on inhibitory control’s impact on uncertainty. If inhibitory control affects the behavioral manifestation of uncertainty, then current Experiment 2 should replicate results of Experiment 1. Conversely, if inhibitory control improves the behavioral manifestation of uncertainty only when overcoming a dominant action tendency, then current Experiment 2 would not yield any significant effects in visual search as a function of differential control levels in the Stroop task.

### 2.2. MATERIALS AND METHODS

#### 2.2.1. Participants

Forty-six healthy participants (recruited via the university’s online experiments system) participated for course credit or a small monetary amount. Recruitment criteria were identical to current Experiment 1. Two participants were excluded from further analysis due to having extreme number of errors (i.e., above 2.5 standard deviations from the mean). Thus, 44 participants were included in the following analyses (32 females and 12 males, average age = 23.41 years, *SD* = 3.95).

#### 2.2.2. Apparatus

Identical to Experiment 1.

#### 2.2.3. Procedure

The experimental procedure was identical to Experiment 1, except for modifying proportions of the visual search task so that target-present and target-absent trials were presented an equal number of times. Accordingly, there were 60 trials of the four possible combinations of the Stroop and visual search tasks; namely, congruent Stroop trials followed by target-present visual search trials, congruent Stroop trials followed by target-absent visual search trials, incongruent Stroop trials followed by target-present visual search trials and incongruent Stroop trials followed by target-absent visual search trials.

#### 2.2.4. Statistical analysis

Statistical analyses were identical to those described in current Experiment 1. A power analysis using G*Power 3.1 (Faul et al., 2007) based on the previous effect sizes (Kalanthroff et al., 2016) indicated that the current sample allowed for examination of the two-way interaction between inhibitory control and uncertainty at a power >95% to test medium to large size effects with a Type 1 error (α < 0.05).

### 2.3. RESULTS

**Figure 3:**
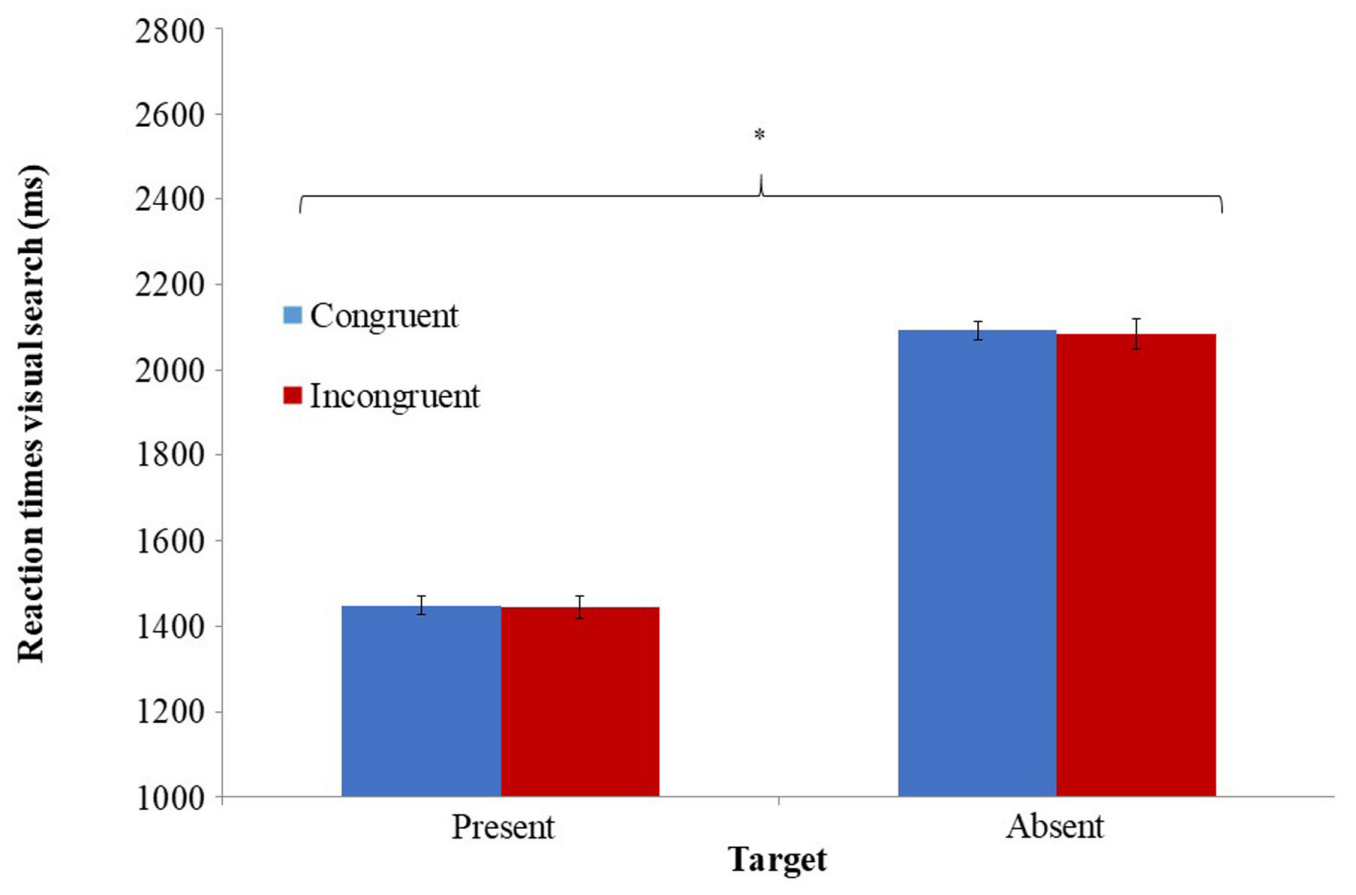
Mean RT in the different conditions of the visual search task as a function of the different inhibitory control conditions. * Significant at *p* < .0001 levels. Error bars represent ± 1 standard error (Cousineau, 2005).

As a manipulation check, we analyzed performance in the Stroop task. RTs in the Stroop task significantly differed as a function of trial type *t*(43) = 6.21, *p* < .0001, *η^2^_p_* = .47, with longer RTs in incongruent trials (M = 634 ms, SD = 177) compared to congruent trials (M = 591 ms, SD = 154). Accuracy in the Stroop task did not significantly differ for congruent (M = .96, SD = .04) and incongruent trials (M = .95, SD = .05), *t*(43) = 1.6, *p* = .12, *η^2^_p_* = .06. These results indicate that congruent and incongruent Stroop induced different levels of inhibitory control. With respect to our a-priori hypothesis regarding interaction between inhibitory control and uncertainty, as expected, the two-way ANOVA revealed a significant main effect for uncertainty *F*(1, 43) = 214, *p* < .0001, *η^2^_p_* = .83, indicating slower RTs to target-absent trials (M = 2088 ms, SD = 494) compared to target-present trials (M = 1446 ms, SD = 290). No significant interaction was found between inhibitory control induction and uncertainty in RTs of the visual search task *F*(1, 43) = .04, *p* = .84. The planned comparisons did not detect any significant influence of inhibitory control induction on RTs, *F*(1, 43) = .4, *p* = .53, *F*(1, 43) = .05, *p* = .82 for target-absent and target-present trials respectively (***Figure 3***). An identical two-way ANOVA with visual search accuracy revealed a significant main effect for uncertainty level *F*(1, 43) = 71.69, *p* < .0001, *η^2^_p_* = .63, with higher accuracy for target-absent trials (M = 98%, SD = 1.8) compared to target-present trials (M = 89%, SD = 7.3). There was no significant interaction between inhibitory control induction and uncertainty level on accuracy *F*(1, 43) = .033, *p* = .857.

Subjective certainty of participants was significantly reduced in target-absent trials (M = 70.66, SD = 16.65) compared to their subjective certainty in target-present trials (M = 91.69; SD = 8.38), *F*(1, 43) = 59.24, *p* < .0001, *η^2^_p_* = .57 (*Figure S1*).

### 2.4. DISCUSSION

Results of Experiment 2 suggest that inhibitory control induction affects behavioral manifestation of uncertainty only under certain conditions: specifically, inhibitory control improved behavioral responses to uncertainty only when they were rare and unexpected. Without a robust action tendency in the visual search task, effects of inhibitory control induction were not significant. One may contend that modifying the proportions of the visual search trials influenced the level of uncertainty that participants are exposed to. Interestingly, altering proportions of visual-search trials had no effect on participants’ subjective certainty. The question whether inducing inhibitory control always facilitates reaction times when overcoming action tendencies remains. We conducted a third experiment testing whether inhibitory control induction improves any rare-event trial-type, or whether the facilitation observed in Experiment 1 is specific to high-uncertainty trials.

## 3. EXPERIMENT 3

### 3.1. INTRODUCTION

Experiment 3 was designed to test the hypothesis that inhibitory control induction affects visual search trials that require participants to overcome action tendencies. Accordingly, Experiment 3 featured mostly target-absent trials, while having equal proportions of high- and low- inhibitory control induction. If inhibitory control induction facilitates resolution of rare trials, then we should see a facilitation in target-present RTs following an incongruent Stroop trial. However, a different pattern of results would suggest that both uncertainty level and action tendencies play a role in determining the effect of inhibitory control induction on RTs.

### 3.2. METHOD

#### 3.2.1. Participants

Forty-one healthy participants (recruited via the university’s online experiments system) participated for course credit or a small monetary amount. Recruitment criteria were identical to current experiments 1 and 2. Two participants did not complete the task due to technical difficulties, one participant chose to exit the experiment before ending it, and one participant reported focusing her gaze at the bottom of the screen during the Stroop task, effectively avoiding from reading the word, preventing her from activating inhibitory control. Three participants were omitted due to having extreme number of errors (i.e., above 2.5 standard deviations from the mean). Thus, 34 participants were included in the following analyses (21 females and 13 males, average age = 24.09 years, SD = 2.4).

#### 3.2.2. Procedure

The experimental procedure was identical to current experiments 1 and 2. The one difference being an opposite distribution of target-absent trials and target-present trials compared to Experiment 1, so that 80% of the trials featured target-absent trials and only 20% of trials featured target-present trials. Stroop proportions remained equal (i.e., 50% for congruent and for incongruent trials).

#### 3.2.3. Statistical analysis

Statistical analyses were identical to current experiments 1 and 2. A power analysis using G*Power 3.1 (Faul et al., 2007) based on the previous effect sizes (Kalanthroff et al., 2016) indicated that the current sample allowed for examination of the two-way interaction between cognitive control and uncertainty at a power > 95% to test medium to large size effects with a Type 1 error (α < 0.05).

### 3.3. RESULTS

**Figure 4:**
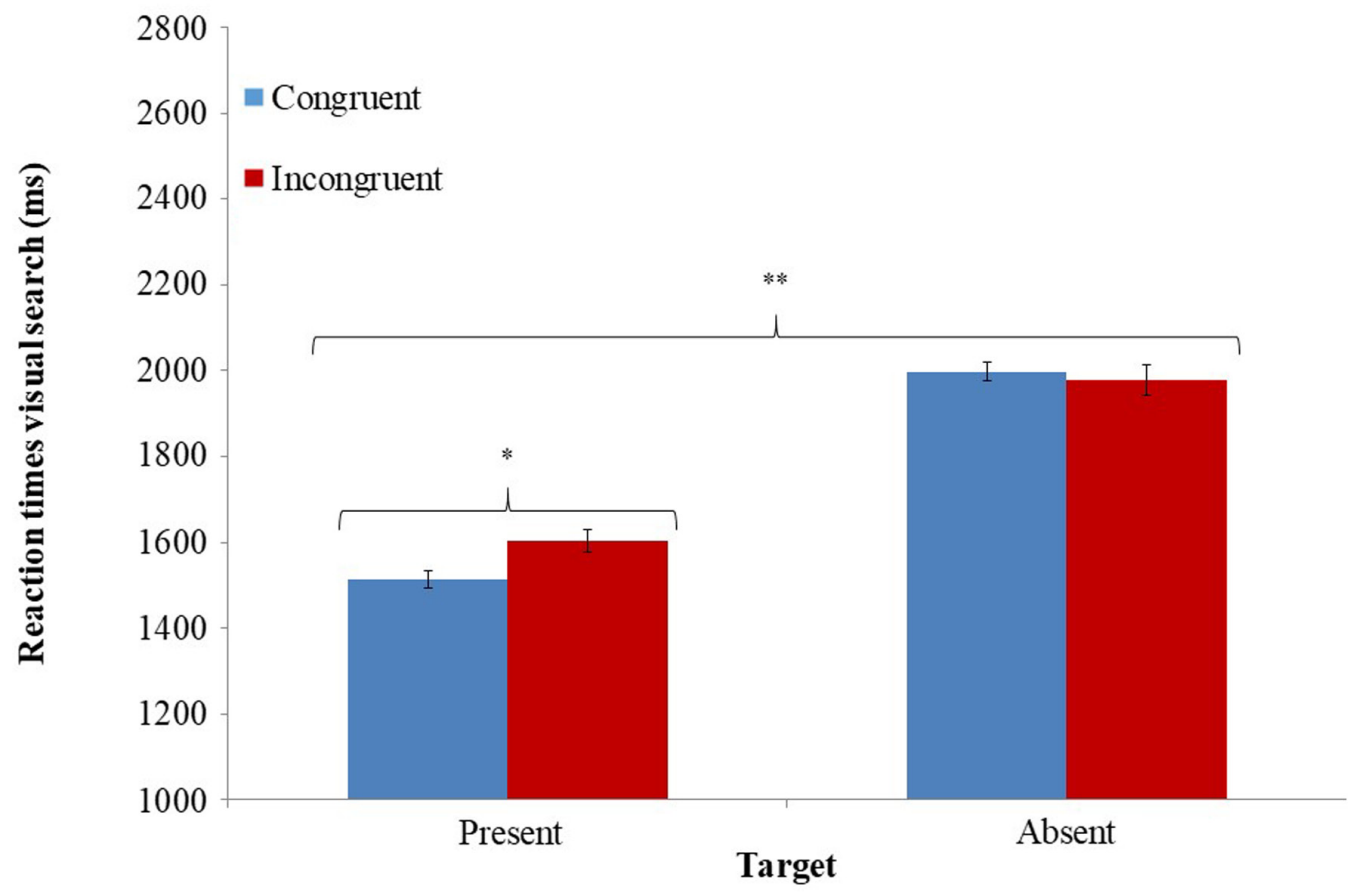
Mean RT in the different conditions of the visual search task as a function of the different inhibitory control conditions. * Significant at *p* < .005 levels; ** Significant at *p* < .0001. Error bars represent ± 1 standard error (Cousineau, 2005).

As a manipulation check, we analyzed performance in the Stroop task. RTs in the Stroop task significantly differed as a function of trial type *t*(33) = 4.97, *p* < .0001, *η^2^_p_* = .43, with longer RTs in incongruent trials (M = 583 ms, SD = 140) compared to congruent trials (M = 549 ms, SD = 125). Accuracy in the Stroop task did not significantly differ for congruent (M = .96, SD = .02) and incongruent trials (M = .95, SD = .04), *t*(33) = 1.76, *p* = .09, *η^2^_p_* = .09. These results indicate that the Stroop task induced different levels of inhibitory control. With respect to our a-priori hypothesis about relations between inhibitory control and uncertainty, as expected a two-way ANOVA revealed a significant main effect for uncertainty level *F*(1, 33) = 95.86, *p* < .0001, *η^2^_p_* = .74, indicating slower RTs to target-absent trials (M = 1987 ms, SD = 431) compared to target-present trials (M = 1559 ms, SD = 327; ***Figure 4***). A significant interaction was found between induction of inhibitory control and uncertainty level *F*(1, 33) = 12.94, *p* < .01, *η^2^_p_* = .28. A smaller, yet significant main effect of inhibitory control on uncertainty was evident *F*(1, 33) = 6.44, *p* < .05, *η^2^_p_* = .16, with incongruent Stroop trials resulting in slower RTs to visual search task (M = 1790 ms, SD = 409) compared to congruent Stroop trials (M = 1756 ms, SD = 393). The two planned comparisons indicated that participants were significantly slower to resolve target-present trials (i.e., low-uncertainty trials) following priming of inhibitory control (i.e., after an incongruent Stroop trial; mean = 1604 ms, SD = 341), compared to resolving target-present trials when participants’ control system was not induced (e.g., after a congruent Stroop trial; M = 1514 ms, SD = 331), *F*(1, 33) = 11.68, *p* < .005. Induction of inhibitory control did not modify resolution of high-uncertainty trials *F*(1, 33) = 2.61, *p* = .12. A similar analysis of participants’ accuracy in the visual search task did not detect effects of inhibitory control induction on uncertainty, *F*(1, 33) = .47, *p* = .49, *η^2^_p_* = .01, and *F*(1, 33) = .37, *p* = .54, *η^2^_p_* = .01 for the two-way interaction and for main effect of inhibitory control induction on uncertainty respectively.

Subjective certainty of participants was significantly reduced in target-absent trials (M = 76.66, SD = 17.39) compared to their subjective certainty in target-present trials (M = 93.75; SD = 7.54), *F*(1, 32) = 33.79, *p* < .0001, *η^2^_p_* = .51 (*Figure S1*; note that one participant did not complete these ratings).

### 3.4. DISCUSSION

Experiment 3 featured the same task (***Figure 1***) with the dominant response being response to high-uncertainty trials (target absent). Here, induction of inhibitory control affected only target-present trials (i.e., low uncertainty) with no detectable effect on target-absent trials (i.e., high uncertainty). Experiment 3’s results are in line with previous findings suggesting that inhibitory control system results in slower reaction times in a following trial – known as post-error slowing (Rey-Mermet & Meier, 2017). While priming’s effect was selective and different from current Experiments 1 and 2, the subjective assessment of certainty resembled Experiments 1 and 2. Therefore, current results suggest that inhibitory control induction affects the non-dominant response while not affecting subjective level of uncertainty. However, while in Experiment 1 high level of inhibitory control resulted in faster RTs in the non-dominant response, in Experiment 3 high level of inhibitory control resulted in slower RTs in the non-dominant response. This point will be elaborated in the general discussion.

## 4. GENERAL DISCUSSION

Our three experiments examined effects of momentary inhibitory-control induction on the behavioral manifestation of uncertainty in changing contexts. This is the first study demonstrating inhibitory control’s impact on behavioral manifestation of uncertainty within a single trial, mimicking the vicious cycle – a seminal component in clinical theories connecting unwanted thoughts with unwanted behaviors (Salkovskis, 1985). In the condition predominated by target-present trials (i.e., low-uncertainty trials; Experiment 1), inducing inhibitory control selectively shortened resolution of target-absent trials (i.e., high-uncertainty trials). The same induction did not affect performance in target-present or target-absent trials when both were equally likely to appear (Experiment 2). Finally, in the condition predominated by target-absent trials (i.e., high-uncertainty trials; Experiment 3), inducing inhibitory control did not affect behavioral responses to uncertainty, but instead was associated with slowing responses on target-present trials (low-uncertainty trials).

Cognitive Behavioral Therapy (CBT) conceptualizes psychopathologies riddled with repetitive unwanted thoughts and behaviors (e.g., OCD, health anxiety, body dysmorphic disorder) as stemming from a vicious cycle, in which an unwanted thought induces distress, which a patient tries to alleviate by an behavior (Salkovskis, 1985). These behaviors paradoxically facilitate negative reinforcement, instigating a vicious cycle. Uncertainty is a common theme in these disorders, supposedly driving unwanted maladaptive behaviors. The current study is the first to systematically explore whether we can affect the behavioral manifestation of uncertainty as it arises or irrelevant actions such as the urge to check.

Our results are coherent with predictions of the action tendencies framework (Gibson, 1979). Inducing inhibitory control only affects situations that require overcoming a dominant response or an action tendency (Experiments 1, 3). Reacting to target-absent trials induces more uncertainty and more checking behaviors compared to target-present trials (Toffolo et al., 2013, 2014). Participants’ checking behaviors correlate with RTs during target-absent trials (Toffolo et al., 2013, 2014). In Experiment 1, when the visual-search target was present in most trials and the associated ‘present’ button was the dominant response, inducing inhibitory control (e.g., ‘RED’ written in blue) was associated with resolving the more uncertain condition (target-absent) faster than resolving less uncertain condition. One potential explanation is that resolution of the Stroop conflict reduced the grip of the action tendency to press the ‘present’ button. Notably, participants’ subjective experience remained similar across experiments, suggesting that objective behavioral manifestations of uncertainty may differ from one’s subjective experience.

While we employed a behavioral explanation for current results, one may consider a cognitive framework of expectancies. According to this framework, inducing inhibitory control affects unexpected trials – in Experiment 1, inducing inhibitory control leads to faster RTs in the unexpected target-absent trials, which require resolution of uncertainty. In Experiment 2, where both trial-types appear 50 percent of the time, there is no clear expectation and thus, inhibitory control does not impact RTs.

From an experimental standpoint, the current work adds to the ongoing debate over conflict adaptation (Botvinick et al., 2001; Rey-Mermet & Meier, 2017; Schmidt, 2019; Verguts & Notebaert, 2009). Verguts and colleagues (2011) suggest that post-conflict processes may lead to momentary increased focusing on target-relevant features. Various results expanded this notion to different tasks and domains (Wendt et al., 2012). Our current Stroop and visual search tasks differ from common tasks (Braem et al., 2019) in that the Stroop and visual search tasks feature separate perceptual, spatial and motor features. It is possible that such post-conflict focusing is more evident when overcoming an action tendency or in unexpected trials. Our results support this notion but suggest that the level of uncertainty affects the type of effect. Results of our third Experiment (Exp. 3) fit the prediction of post-conflict slowing as the post-conflict focusing yields slower responses in target-present trials, which are the less frequent. However, when the unexpected experimental condition features high uncertainty (Exp. 1), the same post-conflict processes yield faster reaction times. This opposite directionality suggests that the reduction of RTs in target-absent trials is specific to high-uncertainty conditions. It is plausible that overcoming the urge to check under high uncertainty is facilitated by inhibitory control. When the response is more automatic, as in target-present trials, the same inhibitory-control induction slows down automaticity. Future studies disentangling action tendencies and frequency of high uncertainty trials may shed light on the processes which drive the current effects.

Current results have experimental and clinical implications. Inhibitory control is central in human experience and daily function; from a cognitive perspective, our results stress that studies assessing inhibitory control may benefit from evaluating bottom-up processes such as action tendencies (Perri, 2020). Our results also answer a growing scientific need to experimentally assess and affect uncertainty (Shihata et al., 2016). From a neuroscience standpoint, our results warrant studying the functional and anatomical connections between inhibitory control and uncertainty circuits. Inhibitory control’s neural underpinnings are well delineated in discrete circuits (Robbins et al., 2012; Williams, 2016), while uncertainty’s neural underpinnings require further studies and are context-dependent. Multiple imaging techniques probed uncertainty in response to threat or when facing uncertain economic choices (Bach & Dolan, 2012; Grupe & Nitschke, 2013), providing invaluable knowledge on the role of the reward system and the Bed Nucleus of the Stria Terminalis (BNST) but uncertainty’s core features are debated. Studying mild uncertainty while optimizing our scanning sequences to detect BNST activation and connectivity may improve our understanding of uncertainty, its resolution and its utility in neuro-psychiatric disorders such as OCD (Banasikowski & Hawken, 2019).

For clinicians, our results highlight a way to improve behavioral responses to uncertainty, which could be relevant to multiple psychopathologies involving repetitive, unwanted thoughts and behaviors. An immediate clinical implication relates to OCD. The fact that altering action tendencies produced differential effects integrates top-down and bottom up theories of OCD. Some have suggested that inhibitory control deficits are a biomarker for OCD (Menzies et al., 2008), whereas others suggested that OCD is driven by more basic processes such as action tendencies (Dayan et al., 2017). Our results consolidate these two explanations and indicate that the effects of inhibitory control, a hallmark of regulatory attentional processes, on uncertainty may depend on a basic motor process – action tendencies.

Future studies may test the impact of inhibitory control induction on behavioral manifestations of uncertainty in different patient populations, possibly informing us on unwanted thoughts and behaviors’ converging and diverging pathways (Linkovski, Weinbach, et al., 2019). Others may test our predictions using different trial proportions in our paradigm or test our hypotheses with different uncertainty tasks while inducing differential uncertainty levels. Such tasks might assist in breaking down resolution of uncertainty to sub-components such as decision thresholds (Banca et al., 2014). An alternative possible interpretation of our results is that inducing inhibitory control reduces the urge to check rather than the behavioral manifestation of uncertainty. Our design does not enable us to disentangle this possibility. Therefore, a future study is needed to test whether inducing inhibitory control reduces the urge to engage in behaviors such as checking in other paradigms (Strauss et al., 2020), irrespective of uncertainty levels, thereby dissociating inhibitory control’s effects on the behavioral manifestation of uncertainty and the urge to act. Clinically, future studies may dissociate the behavioral account (i.e., action tendencies) from the cognitive account (i.e., expected uncertainty) and explore whether computerized inhibitory training, under conditions which require overcoming action tendencies, improves resolution of uncertainty or associated behaviors such as checking (Strauss et al., 2020) and whether therapeutic interventions for OCD or Obsessive-Compulsive and Related Disorders modify the association between inhibitory control and behavioral manifestations of uncertainty (Enander et al., 2019; Hirschtritt et al., 2017; Kalanthroff et al., 2018; Lee et al., 2019; Linkovski et al., 2018; Linkovski, Wheaton, et al., 2019; Lochner et al., 2017; Mathews et al., 2018). Neuroimaging studies utilizing our task may reveal the neural basis of the interaction between inhibitory control, uncertainty and action tendencies, since identifying specific uncertainty-related regions may require specific scanning sequences (Theiss et al., 2017).

In conclusion, the current study is the first to provide a detailed account of the relationship between inhibitory control, action tendencies and responses to uncertainty in a clinically-informed experimental design. The patterns of observed results suggest that the effect of inhibitory control on uncertainty is apparent only when action tendencies are accounted for.

## DATA ACCESSIBILITY STATEMENT

Data and materials are available on the Open Science Framework *https://osf.io/gs9hj/*.

## ADDITIONAL FILE

The additional file for this article can be found as follows:

10.5334/joc.133.s1 Figure S1. Subjective ratings of certainty levels as a function of trial type. * Significant at *p* < .0001 levels. Error bars represent ±1 standard error (Cousineau, 2005).
