# Supplementary figures and images for: Momentary Induction of Inhibitory Control and Its Effects on Uncertainty
Source: J Cogn. 2021 Jan 21;4(1):10. doi: 10.5334/joc.133 (PMC7824980; doi:10.5334/joc.133)

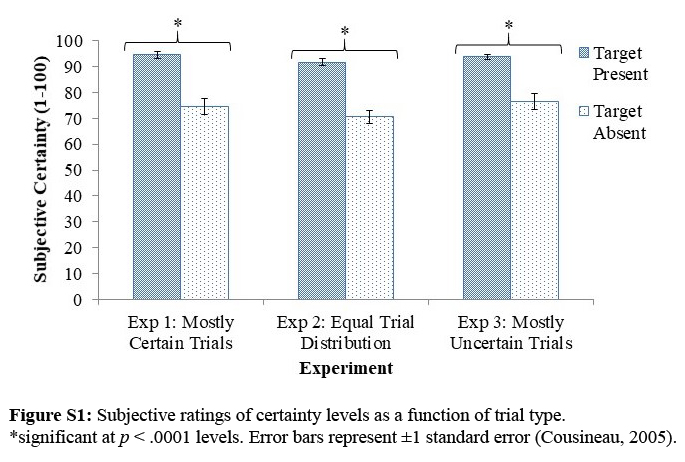

Supplement: Figure S1. — Subjective ratings of certainty levels as a function of trial type. * Significant at p < .0001 levels. Error bars represent ±1 standard error (Cousineau, 2005). [file joc-4-1-133-s1.jpg]
